# Place preference and vocal learning rely on distinct reinforcers in songbirds

**DOI:** 10.1038/s41598-018-25112-5

**Published:** 2018-04-30

**Authors:** Don Murdoch, Ruidong Chen, Jesse H. Goldberg

**Affiliations:** Department of Neurobiology and Behavior, Cornell University, Ithaca, NY 14853 USA

## Abstract

In reinforcement learning (RL) agents are typically tasked with maximizing a single objective function such as reward. But it remains poorly understood how agents might pursue distinct objectives at once. In machines, multiobjective RL can be achieved by dividing a single agent into multiple sub-agents, each of which is shaped by agent-specific reinforcement, but it remains unknown if animals adopt this strategy. Here we use songbirds to test if navigation and singing, two behaviors with distinct objectives, can be differentially reinforced. We demonstrate that strobe flashes aversively condition place preference but not song syllables. Brief noise bursts aversively condition song syllables but positively reinforce place preference. Thus distinct behavior-generating systems, or agencies, within a single animal can be shaped by correspondingly distinct reinforcement signals. Our findings suggest that spatially segregated vocal circuits can solve a credit assignment problem associated with multiobjective learning.

## Introduction

Diverse behaviors can be shaped by primary reinforcement such as reward (e.g. food or water) and punishment (e.g. electric shock), including place preference, lever pressing, action sequencing and timing, reaching, choice tasks, and more^1,2^. Electrical or optogenetic activation of ascending neuromodulators such as dopamine can also reinforce a wide range of actions coincident with the stimulation^3,4^. The diffuse, non-topographic projection patterns of ascending neuromodulatory systems are well-suited to carry reinforcement signals globally to multiple action-generating modules in basal ganglia and cortex^5–7^.

Yet one problem with global reinforcement signals is credit assignment: how does the brain ‘know’ which action caused a reward and, relatedly, which action-generating neural circuit requires synaptic plasticity and associated policy updating to improve performance? Superstitious behaviors acquired during reinforcement learning exemplify how global reinforcement signals can mis-assign credit to a motor act temporally contiguous with, but causally unrelated to reinforcement^8^. Stereotypic body rotations, arm and leg movements acquired during simple tapping or pecking tasks further demonstrate that motor regions controlling arm, leg, and orientation circuits share common, broadcasted reinforcement signals^9,10^.

The credit assignment problem is particularly severe in cases when an agent pursues multiple objectives^11–14^. For example, consider a toddler babbling to herself while stacking blocks. She uses her vocal motor system to speak and her hands and arms to stack. Learning these tasks depends on different types of feedback. Learning to talk may rely on comparison of sensory feedback to an internal auditory target, while learning to stack blocks may rely on comparison of sensory feedback to an entirely independent visual target.

Machine learning provides potential insights into reinforcement learning (RL)^15–17^. Whereas standard RL algorithms optimize a single cost function (e.g. maximize cumulative reward) with a scalar reinforcement signal^18^, in multi-objective learning a single agent can be endowed with independent sub-agents which are trained by an equal number of agent-specific reinforcement signals^15–17^. In the babbling toddler, for example, auditory error signals would reach the vocal motor system (and not the block building one) to shape future vocalizations. Meanwhile errors such as tower collapse would reach the block-building system (and not the vocal motor one) to shape future block building policy^19^. To our knowledge it remains unknown if a single animal possesses distinct ‘agencies’ inside its brain which are, by definition, shaped by agent-specific reinforcement signals.

Here we use songbirds to test if an animal can compute behavior-specific reinforcement signals and route them to the corresponding behavior-producing parts of the motor system. Songbirds sing and navigate (i.e. hop and fly). An objective of the song system is to produce a target sequence of sounds derived from the memory of a tutor song^20–22^. An objective of a navigation system is to avoid aversive stimuli^23^. Song learning can be reinforced with distorted auditory feedback (DAF): if a brief broadband sound is played to a bird as it sings a target syllable a certain way, the bird modifies its song to avoid the feedback^24,25^. A song-relevant reinforcement signal thus derives from auditory error^26–29^. Navigation policy can be reinforced with a bright strobe light: if a strobe is flashed in a specific place, many animals learn to avoid that place^30^. A navigation-relevant reinforcement signal can thus derive from an aversive visual stimulus. Confusing these reinforcement signals could be maladaptive, for example if a bird sang a reinforcing song syllable while perched next to a snake nest.

**Figure 1:**
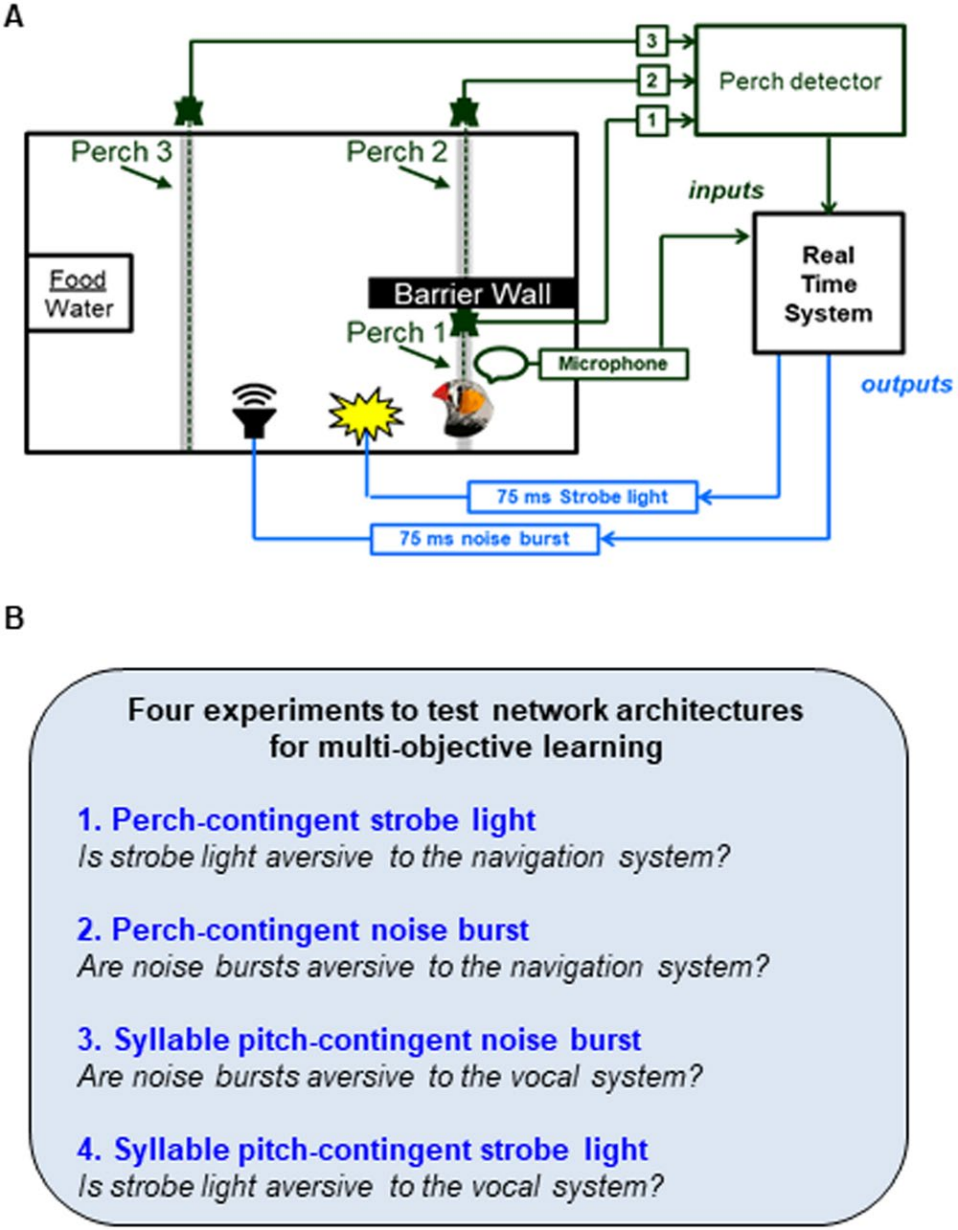
Experimental control of place preference and song syllable learning. (**A**) Schematic of experimental homecage. Signals from perch-mounted IR beam breaks and an overhead microphone provided inputs (green) to a system that analyzed perch occupancy and song features in real time. The system sent outputs (blue) to LEDs for strobe light feedback and to speakers for noise burst feedback. The system implemented one of four contingencies: (1) Perch contingent strobe light, to test if strobe influences place preference; (2) Perch contingent noise, to test of noise influences place preference; (3) Song syllable pitch contingent noise, to test if noise influences syllable selection; and (4) Syllable pitch contingent strobe, to test if strobe light influences syllable selection.

**Figure 2:**
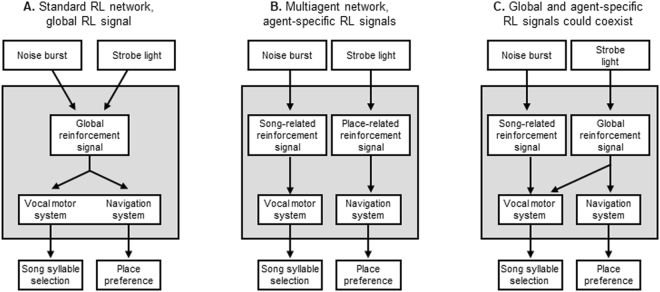
Different network architectures make specific predictions for how multi-objective reinforcement learning is implemented. (**A**) Schematic of a standard RL network where a single reinforcement signal acts globally on multiple parts of the motor system to shape the policy of multiple behaviors. This architecture predicts that both strobe light and noise burst will be aversive to both vocal motor and navigation systems, i.e. will shape both song syllables and place preference. (**B**) A multi-agent RL network where each behavior is shaped by its own behavior-specific reinforcement signal. This architecture predicts that noise will shape song but not place preference, and that strobe will shape place preference but not song. **(C**) Global and behavior-specific reinforcement signals might coexist. Here, it is imagined that strobe light drives reinforcement signals that reach all parts of the motor system, whereas DAF-related reinforcement signals target specifically the vocal motor system. This architecture predicts that DAF will shape song but not place preference, and that strobe will shape both song and place preference.

The ability of songbirds to generate distinct behaviors with distinct objectives presents a unique opportunity to test different network architectures for multi-objective learning. To determine if vocal and place learning can be shaped by shared, overlapping, or distinct reinforcers, we built a closed-loop system that provides either strobe light or noise feedback contingent on zebra finch spatial position or pitch of a target song syllable (Fig. 1). As shown in Fig. 2, distinct learning algorithms require distinct network architectures that make distinct and specific experimental predictions. In a standard RL network with a scalar, global reinforcement signal, both strobe and noise could similarly reinforce both song pattern and place preference (Fig. 2A). In a multi-agent RL architecture where each behavior is independently trained by a behavior-specific reinforcement signal, noise could reinforce song pattern but not place preference, and strobe could reinforce place preference but not song pattern (Fig. 2B). Finally, global and target-specific reinforcement signals might coexist: one of the stimuli could drive a global error signal that reinforces both behaviors, while another could specifically target one behavior (Fig. 2C).

We find that song pattern and place preference are differentially reinforced by sound and strobe light respectively, consistent with multi-agency. Our results provide support for animal implementation of a specific network architecture used in machine learning and suggest a logic for the spatial segregation of vocal motor circuits that independently evolved in diverse vocal learning species^17,31^.

## Results

**Figure 3:**
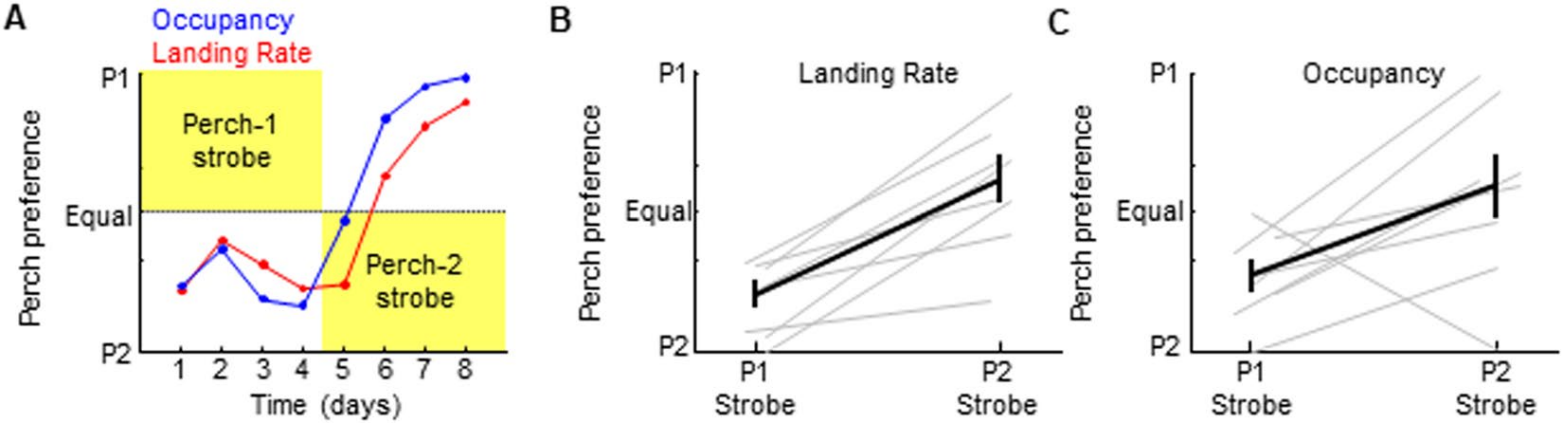
Strobe light is aversive to the navigation system. (**A**) Perch occupancy (blue) and landing rate (red) on test perches from an example bird, plotted over four days of perch 1-contingent strobe (P1 Strobe), followed by four days of perch 2-contingent strobe (P2 Strobe). (**B**,**C**) Average landing rates (**B**) and Occupancies (**C**) for eight birds across P1- and P2- contingent strobe conditions demonstrate preference for non-strobed perch. Ordinates in (**B**,**C**) are the probability of P1 ranging from 0 to 1.

To test if strobe light drives place learning, we implemented perch-contingent strobe light feedback: if a bird landed on one of two ‘target’ perches, a 75 millisecond strobe light stimulus discharged at the moment of landing and then continuously at 2 ± 0.25 Hz for as long as the perch was occupied (see Methods). Birds avoided the strobe-associated perch (Fig. 3). Perch 1-contingent strobe resulted in preference for perch 2 (Perch 2 landing rate: 81.3 ± 11.7%, p < 0.0001; Perch 2 occupancy: 73.7 ± 14.6%, p < 0.01, one-sample t tests, n = 6 birds). Contingency reversal with perch 2-contingent strobe biased preference towards perch 1 (Perch 1 landing rate increased from 18.7 ± 11.7% to 59.4 ± 21.4%, p < 0.001; perch 1 occupancy increased from 26.3 ± 14.6% to 73.7 ± 14.6%, p < 0.05; two-sample t-tests). These data indicate that strobe light negatively reinforces place preference in zebra finches.

**Figure 4:**
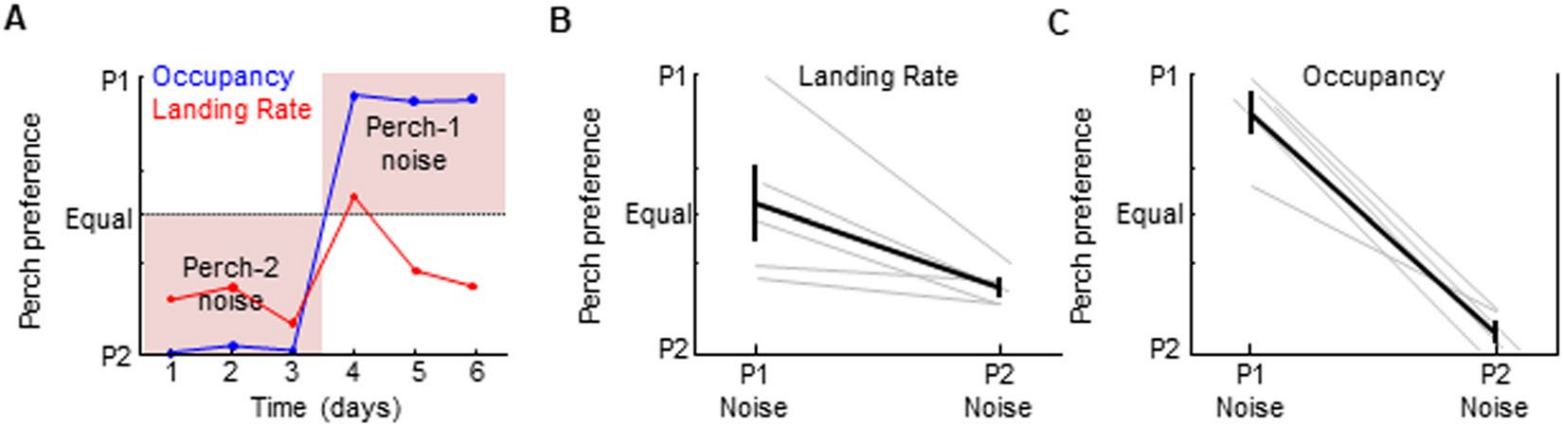
Noise bursts are positively reinforcing to the navigation system. (**A**) Perch occupancy (blue) and landing rate (red) on test perches from an example bird, plotted over three days of perch 2-contingent noise, followed by three days of perch 1-contingent noise. (**B**,**C**) Average landing rates (**B**) and Occupancies (**C**) for five birds across P1- and P2- contingent noise conditions demonstrate preference for the ‘noisy’ perch.

Perch-contingent auditory feedback was implemented exactly as described above except the 75 millisecond strobe was replaced with a 75 millisecond song-syllable like sound played at 88 decibels (dB), less than the average peak loudness of zebra finch song (Methods). Surprisingly, birds acquired a place preference for whichever perch triggered the noise (Fig. 4). Perch 1-contingent noise resulted in preference for perch 1 (Perch 1 occupancy: 86.0 ± 15.1%, p < 0.001, one-sample t test, n = 5 birds). Contingency reversal with perch 2-contingent noise biased preference towards perch 2 (Perch 2 landing rate increased from 46.7 ± 28.7% to 76.5 ± 6.3%, p < 0.05; perch 2 occupancy increased from 14.0 ± 15.1% to 92.8 ± 7.7%, p < 0.001; two-sample t-tests). These data indicate that brief noise bursts positively reinforce place preference.

**Figure 5:**
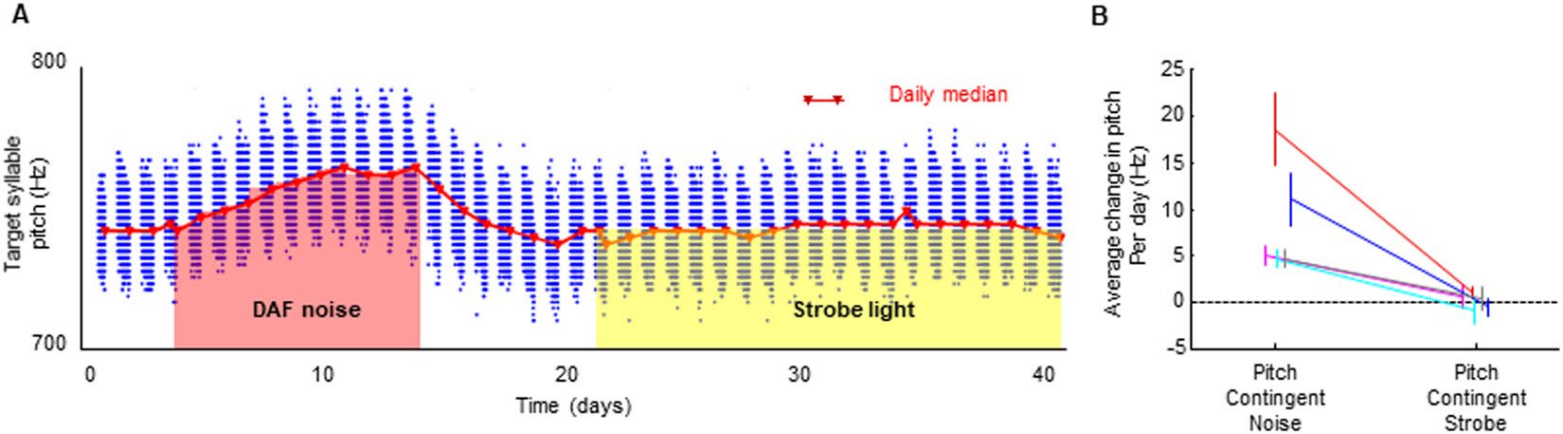
Noise feedback, but not strobe light, drives song syllable learning. (**A**) Blue dots denote mean pitch of target syllable renditions sung over 41 days for one bird. Pink and yellow shading demarcate syllable renditions that triggered noise and strobe light, respectively. (**B**) Average change in target syllable pitch per day during pitch-contingent noise (left) or strobe light (right) (n = 5 birds, errorbars, S.E.M.).

We next carried out song syllable pitch-contingent auditory feedback. In each bird, we chose a ‘target’ harmonic syllable amenable to real-time pitch computation (Methods)^24,25^. After at least three days of obtaining baseline target syllable pitch distributions, we implemented pitch-contingent noise feedback by playing the 75 millisecond noise burst (used in perch preference experiments) during low pitch target syllable variants (Fig. 5). All birds increased the pitch of their target syllable to avoid the noise (average change in pitch per day per bird: 8.8 ± 2.7 Hz, p < 0.05 in 5/5 birds, one-sample t tests), consistent with previous studies^24–26,32–35^. Thus the same noise that was positively reinforcing to the navigation system was aversive to the vocal motor system.

To test if strobe light is aversive to the vocal motor system, we implemented pitch-contingent strobe feedback, exactly as described above except the 75 millisecond sound was replaced with the 75 millisecond strobe stimulus. Birds did not change the pitch of their target syllables to avoid strobe, even when they were given extended periods of time to allow for potentially slower learning (average change in pitch per day per bird: 0.19 ± 3.3 Hz, p > 0.5 in 5/5 birds, one-sampled t tests). In all birds tested, daily pitch-shift was significantly greater during auditory compared to light feedback (Fig. 5B). Thus, the light stimulus that was aversive to the navigation system was not detectably aversive to the song system.

**Figure 6:**
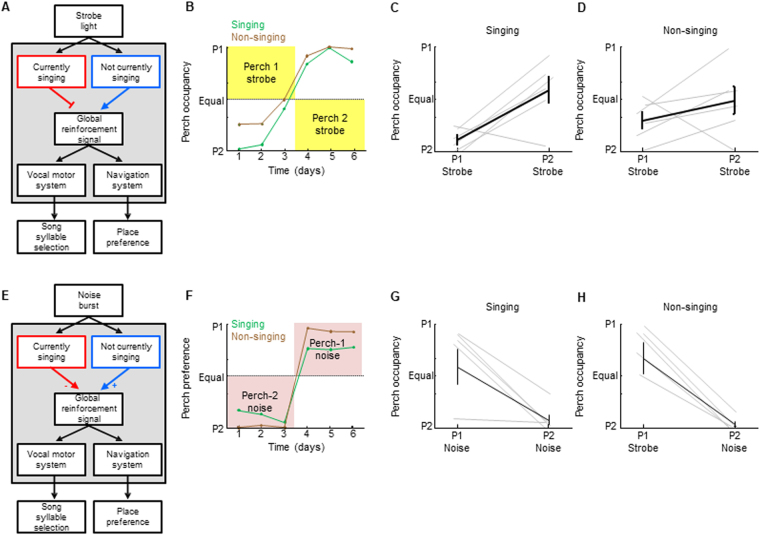
The reinforcing properties of noise and light do not depend on behavioral context. (**A**) A network architecture in which the access of strobe light to a global reinforcement signal is gated by singing state. This architecture would predict that strobe is not aversive when birds are singing. (**B**) Perch occupancy during singing (green) and non-singing (brown) on test perches from an example bird, plotted over three days of perch 1-contingent strobe (P1 Strobe), followed by three days of perch 2-contingent strobe (P2 Strobe). **(C**,**D**) Average perch occupancies during singing (**C**) and non-singing (**D**) for six birds across P1- and P2- contingent strobe conditions demonstrate preference for non-strobed perch during both singing and non-singing periods. (**E**) A network architecture in which the access of noise burst to a global reinforcement signal is gated by singing state. This architecture predicts that noise valence becomes negative during singing such that birds would not choose to sing on ‘noisy’ perches. (**F**) Perch occupancy during singing (green) and non-singing (brown) on test perches from an example bird, plotted over three days of perch 2-contingent noise, followed by three days of perch 1-contingent noise. (**G**,**H**) Average perch occupancies during singing (**G**) and non-singing (**H**) for six birds across P1- and P2- contingent noise conditions demonstrate preference for the noisy perch during both singing and non-singing periods.

The routing of error signals to distinct parts of the motor system could in principle be gated by behavioral context. For example, the noise sound could be aversive during singing but not during non-singing periods (Fig. 6E). To test this, we separately analyzed perch occupancy patterns for singing and non-singing periods during the perch-contingent noise experiments. Birds preferred the ‘noisy’ perch during both singing and non-singing periods (Fig. 6F–H) (Two-way ANOVA showed significant effect of noise on perch occupancy [F(1,19) = 67.67, p < 0.001], and no effect of singing state [F(1,19) = 4.27, p > 0.05] or interaction between noise and singing state [F(1,19) = 1.12, p > 0.]). Thus context-dependent gating of noise aversiveness cannot explain birds’ preference for occupying ‘noisy’ perches.

Similarly, the strobe light might be globally aversive but only during non-singing periods, for example if birds simply did attend to light during singing (Fig. 6E). To test this, we separately analyzed perch occupancy patterns for singing and non-singing periods during the perch contingent strobe experiments. Birds avoided the strobed perch during both singing and non-singing (Fig. 6F–H) (Two-way ANOVA showed significant effect of strobe on perch occupancy [F(1,24) = 15.26, p < 0.001], and no effect of singing state [F(1,24) = 0.23, p > 0.6] or interaction between strobe and singing state [F(1,24) = 2.82, p > 0.1]). Thus context-dependent gating of strobe aversiveness cannot explain place preference for non-strobed perches.

## Discussion

**Figure 7:**
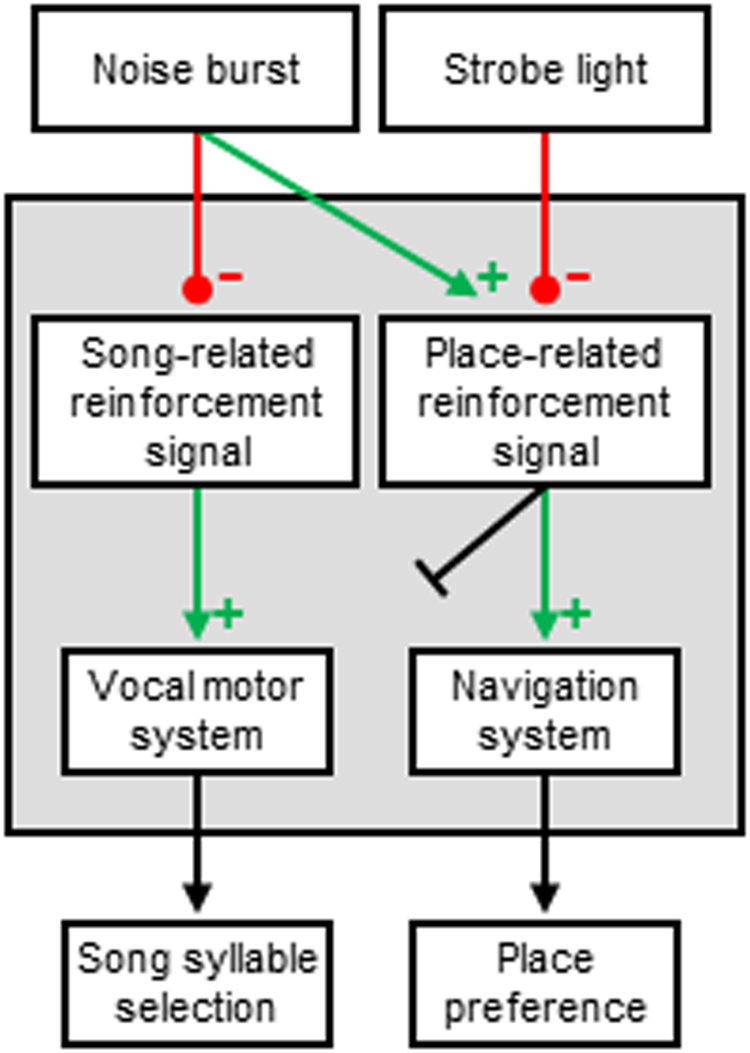
Network architecture supported by our experimental results. Noise burst was aversive to the vocal motor system and was reinforcing to the navigation system. Strobe light was aversive to the navigation system but was apparently unable to access vocal motor circuits.

Vocal learning poses unique problems because vocalizations are often produced as animals are doing other things. Toddlers babble even as they learn to walk; birds learn to sing even as they hop and fly around an environment. In machines, one way to solve the credit assignment problem associated with multi-objective reinforcement learning is to endow an agent with independent sub-agents which are trained by an equal number of agent-specific reinforcement signals^15–17^. We report that song and place learning are driven by distinct reinforcers, demonstrating that action-specific reinforcement signals can be computed and precisely routed to the corresponding action-generating parts of the motor system (Fig. 7). Thus a single zebra finch is endowed with multiple agencies.

Our results provide a clear counterexample to general purpose models of learning that rely on global reinforcement^2,5^. The strobe and noise stimuli were not ‘generally’ aversive or reinforcing because vocal and navigation systems responded differently. Multi-agency could arise from specific evolutionary histories endow animals with genetic constraints on the associativity of actions with outcomes^36^. For example, dogs struggle to learn to yawn for food, trapped cats readily learn to escape a cage by pressing a lever but not by grooming, rats associate sounds and lights with electric shock but not with nauseating food, and pigeons can learn to peck a key for food and take flight to avoid a shock, but not vice versa^1,37–39^. The pairing of specific actions with valent consequences in a laboratory setting may be so unnatural that an animal is unable, or ‘contraprepared’, to associate them^40^. In our experiments, it was likely natural for bird to navigate away from a threatening stimulus, but not to avoid eliciting it by singing in a different way. Finally, it may also be natural for a social animal like a zebra finch to navigate towards noisy places and away from quiet ones, as silence may indicate isolation and an associated increased predation risk.

While reinforcing vocalizations based on auditory, but not visual feedback, may be more natural for song imitation^41^, our specific findings of strobe’s lack of an effect on zebra finch pitch learning does not rule out visual access to song systems more generally. Unpredicted strobe lights have previously been shown to interrupt singing, presumably by startle effect^42^, and can also induce a heightened arousal state that enhances memorization of a tutor song^43^. In cowbirds, visual displays by females appear to reinforce specific male vocalizations^44^.

Our findings may shed light on the longstanding mystery of why vocal learning circuits repeatedly evolved to be segregated from other parts of the motor system. Specifically, vocal learning evolved independently in humans, songbirds, hummingbirds and parrots^45–47^. Birds have specialized ‘song systems’ dedicated to vocal learning but not to other behaviors such as grooming, eating, or flight^48–52^. Similarly, humans have specific vocal motor circuits, including Broca’s area, dedicated to speech but not to other orofacial behaviors such as chewing, licking or facial expressiveness^53,54^. A segregated vocal circuit could provide a discrete target for vocalization-specific reinforcement signals that would not contaminate non-vocal behaviors. Absent target-specific reinforcement, several credit assignment problems arise. First, global reinforcement relies on the temporal contiguity of action and outcome: only the neurons whose activity drives an action will be eligible for reinforcement-modulated plasticity^18,27,55^. Yet temporal credit assignment alone may be maladaptive, for example if a babbling bird hits the right note right while perched next to a predator. Standard RL algorithms deploy repetition to make global reinforcement workable: a reinforcement signal of ambiguous attribution on a single trial will, on average, follow the activity of the reinforcement-causing action. Here, much depends on the allowable delays between action and reward, and it thus matters that vocal and place learning pose very different temporal credit assignment problems. During foraging for food or liquid rewards, several seconds of behavior preceding reward can be reinforced^2,10^, which may be commensurate with latencies between foraging decisions and reward receipt as well as the synaptic eligibility trace measured for dopamine-modulated corticostriatal plasticity in ventral striatum^56^. In contrast, the auditory feedback from self-generated vocalizations is almost instantaneous. In songbirds the associability of vocal output and reinforcing auditory feedback was measured at less than 100 milliseconds^24^. We hypothesize that spatially segregated vocal circuits could further enable vocal learning by implementing synaptic plasticity with narrower time windows specialized for the brief delays between vocal variation and valent auditory feedback^24,27,57^. A precedent for brain region-specific time windows for synaptic plasticity has recently been identified in different cerebellar domains mediating different behaviors^58^; it remains unknown if a similar principle could operate in different striatal domains.

What are the precise neural circuits that connect an aversive light flash to the navigation system to drive avoidance behavior, and a song-like noise to the vocal motor system to change syllable pitch? The anatomical segregation of vocal circuits might create a discrete spatial target for song-specific reinfrocement signals. For example, we recently identified song-related auditory error signals in dopaminergic neurons of the songbird ventral tegmental area (VTA)^26^. Using antidromic and anatomical methods we discovered that only a tiny fraction (<15%) of VTA dopamine neurons project to the vocal motor system - yet these were the ones that encoded vocal reinforcement signals. The majority of VTA neurons which project to other parts of the motor system did not encode any aspect of song or singing-related error. This specific ‘song evaluation channel’ embedded inside the ascending mesostriatal dopamine system thus targets singing-related error signals specifically to vocal motor, and not navigation, circuits. Interestingly, many VTA neurons, especially those that do not project to Area X, were activated by noise bursts in that study^26^. If these VTA neurons project to parts of the brain that control navigation policy, then these noise-induced activations could provide a neural correlate of the reinforcing properties of noise that induced place preference.

## Methods

### Animals

Subjects were 11 adult male zebra finches singly housed in behavior boxes singing undirected song. All experiments were carried out in accordance with NIH guidelines and were approved by the Cornell Institutional Animal Care and Use Committee.

### Pitch-contingent, syllable-targeted distorted auditory feedback

In five birds singing undirected song, song was recorded with AT803 Omnidirectional Condenser Lavalier Microphones amplified through a MIDAS xl48 8-Channel Microphone Pre-Amp connected to a National Instruments 6341 data acquisition card at 40 kHz using custom LabVIEW Software running on a windows PC (Dell Optiplex 7040 MT). The distorted auditory feedback (DAF) was a 75 millisecond duration broadband sound bandpassed at 1.5–8 kHz, the same spectral range of zebra finch song^25^. Sound feedback was supplied as 16 bit 44.1 kHz wave file snippets using the Digilent High Performance Analog Shield (Digilent Part #410-309) through Logitech S120 Desktop Speakers. The amplitude was measured with a decibel meter (CEM DT-85A) and maintained at 88 dB, less than the average peak loudness of zebra finch song^59^. Specific syllables were targeted either by detecting a unique spectral feature in the previous syllable (using Butterworth band-pass filters) or by detecting a unique inter-onset interval (onset time of previous syllable to onset time of target syllable) using the sound amplitude as previously described. In both cases a delay ranging from 10–200 ms was applied between the detected song segment and the precise part of the harmonic stack targeted for pitch-contingent DAF. We first determined the baseline pitch of each bird’s target harmonic syllable by recording song without distortion for at least 5 days. The pitch measured by taking a fast Fourier transform of a six millisecond segment within a specified portion of the harmonic stack^32^. The median pitch of the target syllable during day 5 of the baseline period was used as the initial threshold for feedback. On the first day of pitch-contingent DAF (day 6) we distorted target syllable renditions with pitch lower than this threshold. The distortion began 0–2 ms after the 6 ms window used for pitch measurement. Thresholds were automatically updated every 400 renditions if the median pitch of the last 400 renditions was higher than the previous threshold. We continued this protocol for several days until the birds moved their pitch up by at least 40 Hz from baseline (‘up’ days).

### Pitch-contingent, syllable targeted strobe light feedback

After pitch contingent distorted auditory feedback was demonstrably effective in inducing pitch changes, birds were given a zero-feedback epoch of at least 10 days during which their pitch distributions returned to baseline, as previously reported. Then pitch contingent syllable targeted light feedback was conducted exactly as described above, targeting the same syllables in the same five birds, except instead of playing the 75 ms DAF sound a 75 ms strobe light stimulus was flashed. Light feedback was delivered via custom LED panels with 24 LED’s per panel, 2 panels mounted on either end of each perch in a sandwich configuration (35000mcd per LED, manufacturer part #: LED Optek OVLEW1CB9, digikey part # 365-1177-ND). A single strobe event lasted 75 ms. It consisted of 5 milliseconds LEDs on, 65 ms LEDs and cage lights off, followed by 5 ms of LED on, followed by cage lights back on.

### Perch contingent DAF or strobe feedback

Six birds were taken from the colony and placed isolated in the test cages for 6–8 days of perch contingent strobe feedback (3–4 days per perch). The same birds were returned to the colony for at least 1 week and returned to test cages for 6–8 days of perch contingent noise (3–4 days per perch). Each perch was equipped with two 5 mm IR-beam break sensors (Adafruit, product ID: 2168). Beam-break data was acquired and analyzed alongside the microphone signal with an arduino and custom labview code that communicated with either a speaker or strobe lights. Perch landing rate and perch occupancy were calculated for the entire period of the experiment, ruling out lights-off (sleep) periods when birds do not move. The landing reliably caused a beam break independent of where on the perch the bird landed because two IR beams were projected parallel to the surface of the perch, along its entire length. Depending on the contingency, a targeted perch was associated with light or noise feedback by triggering the 75 ms duration noise (or strobe) stimulus 1 millisecond after perch landing and then continuously at 2 ± 0.25 Hz thereafter, for as long as the bird stayed on the perch.

### Statistical analyses

Statistics were first performed with two-way ANOVAs to test for effect of condition (strobe or no strobe, noise or no noise) and singing state (singing and non-singing), followed up with post hoc one-sample t tests to test whether specific conditions differed from the null hypothesis that perches would be equally occupied and landed on. The currently singing state was defined as the period of time from 1 second of silence before a song syllable onset until 1 second of silence after a song syllable offset. The currently non-singing state was all other time (excluding night time as described above).
